# Modifizierte Delphi-Studie zur Implementierung von Early Warning Scores

**DOI:** 10.1007/s00063-025-01336-y

**Published:** 2025-10-01

**Authors:** Peter Nydahl, Florian Grossmann, Vanessa Franke, Marie-Madlen Jeitziner, Susanne Krotsetis, Koroush Kabir, Reto Lingenhag, Tobias Melms, Felix Jakob Neuenfeldt, Dominika Oroszy, Frida Regner, Jan-Peter Braun

**Affiliations:** 1 Pflegewissenschaft und -entwicklung, Universitätsklinikum Schleswig-Holstein, Haus V40, Arnold-Heller-Str. 3, 24105 Kiel, Deutschland; 2 Institut für Pflegewissenschaft und -praxis, Paracelsus Medizinische Privatuniversität, Salzburg, Österreich; 3 Departement Akutmedizin, Universitätsspital Basel, Basel, Schweiz; 4 Pflegewissenschaft und Praxisentwicklung, Universitätsmedizin Frankfurt, Frankfurt am Main, Deutschland; 5 Direktion Pflege, Universitätsspital Bern (Inselspital), Universität Bern, Bern, Schweiz; 6 Pflegewissenschaft und -entwicklung, Universitätsklinikum Schleswig-Holstein, Lübeck, Deutschland; 7 Klinik für Unfallchirurgie, Orthopädie und Sportmedizin, Helios Universitätsklinikum Wuppertal, Wuppertal, Deutschland; 8 Qualitätsmanagement, Kantonsspital Winterthur, Winterthur, Schweiz; 9 Pflegequalitätsentwicklung, Universitätsmedizin Greifswald, Greifswald, Deutschland; 10 Institut für Rettungs- und Notfallmedizin, Universitätsklinikum Schleswig-Holstein, Kiel, Deutschland; 11 Qualitätsabteilung, Universitätsklinikum Ljubljana, Ljubljana, Slowenien; 12 Universitäts Kinder- und Frauenzentrum, Universitätsklinikum Carl Gustav Carus Dresden, Dresden, Deutschland; 13 Klinik für Anästhesiologie, Intensivmedizin und Schmerztherapie, Martin Luther Krankenhaus, Berlin, Deutschland

**Keywords:** Konsensusverfahren, Vitalparameter, Implementierung, Krankenhaus, Patientensicherheit, Consensus method, Vital signs, Hospital, Implementation, Patient safety

## Abstract

**Hintergrund:**

Early Warning Scores (EWS) gelten als effektives Instrument zur frühzeitigen Erkennung klinischer Verschlechterungen im Krankenhaus. Ihre Implementierung stellt jedoch komplexe Anforderungen an Strukturen, Prozesse und Kultur der Einrichtungen.

**Methode:**

Im Rahmen einer modifizierten Delphi-Studie wurden Experten aus dem Netzwerk Initiative Qualitätsmedizin mit praktischer EWS-Erfahrung eingeladen, notwendige Strukturen, Prozesse und typische Fallstricke in den Projektphasen Vorbereitung, Implementierung, Evaluation und Verankerung zu benennen. In 3 Runden wurden Empfehlungen zu den Phasen gesammelt, thematisch geclustert und bewertet (0–10, 10 = max. relevant). Abschließend wurden die Empfehlungen in einem Konsensustreffen konsolidiert.

**Ergebnisse:**

Insgesamt nahmen 8 Personen an allen Runden teil. Aus 505 Antworten wurden 51 konsolidierte Empfehlungen identifiziert, die in allen Phasen als hochrelevant (≥ 7 Punkte) bewertet wurden. Die höchste Priorität erhielten die Schulung aller Berufsgruppen (Mittelwert [MW] 9,3) sowie eine transparente Kommunikation von Ergebnissen und Maßnahmen (MW 9,0). Als häufige Barrieren wurden mangelnde IT-Anbindung, Verantwortlichkeiten und Rückmeldungen genannt. Erfolgsentscheidend sind interdisziplinäre Projektteams, die frühzeitige Evaluation und die Einbettung in klinische Routinen.

**Schlussfolgerungen:**

Die konsensbasierten Empfehlungen können Entscheidungsträger bei der systematischen Einführung von EWS unterstützen. Eine strukturierte Projektplanung, interprofessionelle Schulung und kontinuierliche Rückkopplung sind zentrale Erfolgsfaktoren. Um EWS nachhaltig zu etablieren, sollten sie verbindlich in Qualitätssysteme, Fortbildungen und klinische Routinen integriert werden.

**Zusatzmaterial online:**

Zusätzliche Informationen sind in der Online-Version dieses Artikels (https://doi.org/10.1007/s00063-025-01336-y) enthalten.

## Abstract (en)

**Background:**

Early warning scores (EWS) are considered an effective tool for the early detection of clinical deterioration in hospital settings. However, their implementation poses complex demands on institutional structures, processes, and culture.

**Methods:**

As part of a modified Delphi study, experts from the Initiative Qualitätsmedizin network with practical experience in EWS implementation were invited to identify necessary structures, processes, and common pitfalls across the project phases of preparation, implementation, evaluation, and anchoring. In three rounds, recommendations were collected, thematically clustered, and rated (0–10, 10 = maximally relevant). Finally, the recommendations were consolidated in a consensus meeting.

**Results:**

Eight participants completed all rounds. From 505 responses, 51 consolidated recommendations were identified, all considered as highly relevant (≥ 7 points) in the various rounds. Top priorities were the training of all professional groups (mean 9.3) and transparent communication of results and actions (mean 9.0). Common barriers included lack of information technology (IT) integration, unclear responsibilities, and insufficient feedback. Key success factors were interdisciplinary project teams, early evaluation, and embedding into clinical routines.

**Conclusion:**

The consensus-based recommendations can support decision-makers in the systematic implementation of EWS. Structured project planning, interprofessional training, and continuous feedback are key success factors. For sustainable integration, EWS should be embedded into quality systems, continuing education, and clinical routines.

Frühwarnsysteme wie der Early Warning Score (EWS) gelten als vielversprechendes Instrument zur Erkennung klinischer Verschlechterungen bei Patienten^1^ im Krankenhaus und als geeignetes Instrument zum klinischen Risikomanagement. Doch ihre erfolgreiche Implementierung erfordert mehr als nur eine technische Einführung – es braucht strukturelle, prozessuale und kulturelle Voraussetzungen. In einer Delphi-Studie mit Experten aus dem Netzwerk der Initiative Qualitätsmedizin (IQM) wurden 51 zentrale Erfolgsfaktoren in 4 Projektphasen identifiziert und priorisiert. Besonders hohe Relevanz zeigten interprofessionelle Schulungsmaßnahmen und eine transparente Kommunikation über Ergebnisse und Maßnahmen. Die Ergebnisse bieten praxisnahe Handlungsempfehlungen für Krankenhäuser, die EWS nachhaltig und wirksam in ihre Versorgungsstrukturen integrieren möchten.

## Einführung

Die EWS sind strukturierte Instrumente zur Früherkennung klinischer Verschlechterungen bei stationären Patienten [15]. Durch die Erhebung und Gewichtung von klinischen Parametern ermöglichen sie ein standardisiertes Eskalationsmanagement [14, 16]. Der National Early Warning Score (NEWS) ist dabei das am häufigsten verwendete System [24]. Ein aktueller Rapid-umbrella-Review identifizierte über 500 Einzelstudien in 44 Übersichtsarbeiten [22]. Die Wirksamkeit ist vor allem in Hochrisikopopulationen (z. B. Notaufnahme, Alterstraumatologie, Post-ICU) nachgewiesen, allerdings ist die Evidenzlage insgesamt heterogen. Der Nutzen hängt stark vom klinischen Setting und der Verfügbarkeit von personellen und technischen Ressourcen ab [22, 29].

### Herausforderungen und Erfolgsfaktoren

Die Implementierung von EWS erfordert strukturelle, technische und personelle Anpassungen [1, 16]. Häufige Barrieren sind fehlende Akzeptanz, unzureichende IT-Infrastruktur und hoher Schulungsaufwand [18]. Gleichzeitig können gezielte Schulungen, klare Kommunikationswege und elektronische Unterstützungssysteme (z. B. Red Flags in Patientenkurven) die Anwendung erleichtern [3, 5, 14]. Die EWS unterstützen die interprofessionelle Kommunikation, bedürfen jedoch einer sorgfältigen Integration in die klinische Routine, um Fehlanwendungen oder Alarmmüdigkeit zu vermeiden [2, 12, 29].

### Empirische Einblicke und Forschungsfrage

Eine Onlineumfrage im Netzwerk der IQM, in der Krankenhäuser aus Deutschland, Österreich, der Schweiz und eins aus Slowenien vertreten sind, zeigt, dass EWS bisher nur begrenzt implementiert sind [21]. Von 480 angeschriebenen Einrichtungen nutzen lediglich 30 EWS, meist auf konservativen Stationen. Der Median der Implementierung lag bei 3,5 (Skala 1–10). Förderlich wirkten engagiertes Personal, klare Schulungskonzepte und technologische Unterstützung. Barrieren waren Akzeptanzprobleme, technische Defizite und Zeitmangel für Schulungen [21].

Daraus ergibt sich die zentrale Forschungsfrage: Welche Strategien zur erfolgreichen Implementierung von EWS empfehlen Mitarbeitende, die bereits praktische Erfahrungen im IQM-Netzwerk gesammelt haben? Die Beantwortung könnte zur flächendeckenden Implementierung und nachhaltigen Verankerung von EWS beitragen.

## Methodik

Zur systematischen Erhebung von praxisrelevanten Empfehlungen zur Implementierung von EWS wurde ein modifiziertes 3‑stufiges Delphi-Verfahren geplant [20, 23]. Ziel war es, strukturelle, prozessuale und potenzielle Fehlerquellen (Pitfalls) entlang der 5 typischen Projektphasen eines Implementierungsvorhabens zu identifizieren und zu priorisieren. Die Studie wurde in Übereinstimmung mit der Deklaration von Helsinki (Version 2024) durchgeführt [33]. Da keine vulnerablen Populationen beteiligt waren, die Teilnahme freiwillig und informiert erfolgte und keine personenbezogenen Gesundheitsdaten erhoben wurden, war ein gesondertes Ethikvotum nicht erforderlich. Die Berichterstattung erfolgt anhand der CREDES-Kriterien ([13]; siehe elektronisches Zusatzmaterial Tab. E3).

### Teilnehmende und Rekrutierung

Zur Teilnahme eingeladen wurden Personen aus dem IQM-Netzwerk, die bereits erfolgreich Erfahrungen mit der Implementierung von EWS in ihren Einrichtungen gesammelt hatten. Die Teilnahme beruhte auf freiwilliger Basis. Ausschlusskriterium war das Fehlen praktischer Implementierungserfahrung. Die Rekrutierung erfolgte über eine gezielte Einladung via IQM. Aufgrund der spezifischen Zielgruppe und des Angebots einer Koautorenschaft bei vollständiger Teilnahme an allen Delphi-Runden lernten sich die Teilnehmenden zu Beginn in einem virtuellen Projektmeeting kennen, die Durchführung der Delphi-Studie erfolgte dann anonym [4].

### Modifiziertes Delphi-Verfahren

In diesem Delphi-Verfahren konnten aufgrund verschiedener Gründe, wie geringe Anzahl der Teilnehmenden und statistische Planung, nicht alle erforderlichen methodischen Verfahren angewendet werden (u. a. 3. Runde, a priori Definition der erforderlichen IQR-Grenze, Kontrolle der Übereinstimmung und Abstimmungsstabilität), daher wurde das Delphi-Verfahren an diese Erfordernisse angepasst [19, 26]. Die Erhebung erfolgte online über die Plattform von SurveyMonkey (Dublin, Irland). Das Delphi-Verfahren umfasste 3 methodische Schritte.

### Runde 1 – Ideensammlung

In der 1. Runde wurden die Teilnehmenden gebeten, aus ihrer Sicht notwendige Bedingungen für eine erfolgreiche Implementierung von EWS zu benennen. Die Abfrage erfolgte differenziert für 3 Dimensionen – Strukturen, Prozesse und Pitfalls – in Bezug auf die 5 typischen Projektphasen:

- Vorbereitung (Ist-Analyse, Zieldefinition),
- Planung (Systemauswahl, Prozessdesign),
- Implementierung (Pilotierung, Schulung),
- Evaluation (Wirkung, Anpassung),
- Verankerung (Roll-out, Verstetigung).

Pro Phase und Dimension konnten bis zu 10 Vorschläge eingereicht werden, sodass maximal 150 Einträge pro Person möglich waren. Die in Runde 1 erhobenen offenen Antworten wurden mithilfe einer KI-gestützten Textanalyse ausgewertet, geclustert und anschließend durch das Forschungsteam validiert.

### Runde 2 – Bewertung und Priorisierung

Alle in Runde 1 eingegangenen Antworten wurden zunächst durch das Studienteam inhaltlich geprüft und in einem mehrstufigen Verfahren ausgewertet. Zunächst erfolgte eine thematische Clusterung in den Kategorien Vorbereitung, Planung, Implementierung, Evaluation und Verankerung. Ergänzend kam eine digitale Textanalyse zum Einsatz, die eine strukturierte Auswertung unterstützte und inhaltlich ähnliche Aussagen zusammenführte. Hierzu wurde eine KI-Anwendung (ChatGPT 4.0, OpenAI, San Francisco, Kalifornien, USA) genutzt und im Prompt angewiesen, redundante Formulierungen zusammenzufassen sowie thematische Muster zu verdichten und in Kategorien zu gruppieren. Alle durch digitale und KI-gestützte Verfahren gewonnenen Ergebnisse wurden anschließend von den Forschenden systematisch auf Plausibilität geprüft, kritisch reflektiert und bei Bedarf angepasst. Dadurch wurde sichergestellt, dass die finale Kategorisierung sowohl technologisch unterstützt als auch durch menschliche Expertise validiert war.

Anschließend wurden die zusammengefassten Vorschläge den Teilnehmenden zur Relevanzbewertung vorgelegt. Die Bewertung erfolgte auf einer 10-Punkte-Likert-Skala (0 = gar nicht relevant bis 10 = maximal relevant). Den Teilnehmenden wurde mitgeteilt, dass nur Vorschläge mit einem Medianwert von ≥ 7 in die nächste Runde übernommen würden. Zur Sicherstellung der inhaltlichen Validität wurden Stichproben einzelner Dimensionen manuell validiert.

### Runde 3 – Konsensbildung

Eine geplante 3. Bewertungsrunde entfiel, da in Runde 2 bereits alle zur Finalisierung vorgesehenen Vorschläge eine Bewertung von ≥ 7 erhalten hatten. Stattdessen wurde ein abschließendes virtuelles Konsensustreffen durchgeführt, bei dem die konsolidierten Ergebnisse vorgestellt und gemeinsam diskutiert wurden. Ziel des Treffens war die Entwicklung von konsensbasierten Handlungsempfehlungen zur Implementierung von EWS in deutschen Krankenhäusern.

### Datenanalyse

Die Auswertung der quantitativen Daten erfolgte deskriptiv mittels JASP 0.19.3 (Universiteit van Amsterdam, Amsterdam, Niederlande). Es wurden Mittelwerte und für Bewertungen Mediane und Interquartilsabstände (IQR) berechnet. Zur inhaltlichen Überprüfung der Konsentierung wurden IQR ≤ 2 angestrebt. Aufgrund der nichterforderlichen 3. Runde wurde auf die Berechnung des Intraklassenkorrelationskoeffizienten zur Überprüfung der Übereinstimmung und Abstimmungsstabilität und verzichtet. Freitextantworten wurden thematisch kodiert und inhaltsanalytisch ausgewertet. Zur Sicherung der Datenqualität wurden alle Antworten auf Konsistenz und Vollständigkeit überprüft.

## Ergebnisse

An der modifizierten Delphi-Studie beteiligten sich 28,6 % der eingeladenen Experten (*n* = 8 von 28). Die Charakteristika der Teilnehmenden sind in Tab. 1 dargestellt. In der ersten Runde wurden insgesamt 505 Einträge durch die Teilnehmenden generiert (Zusatzmaterial, Tab. E1).

**Tab. 1:** Soziodemografische Daten

Item	Prozent (Anzahl), Mittelwerte
Profession	Pflege 62,5 % (*n* = 5), Medizin 25 % (*n* = 2), Qualitätsmanagement 12,5 % (*n* = 1)
Krankenhaustyp	Schwerpunkt- oder Maximalversorgung 87,5 % (*n* = 7), Grund- oder Regelversorger 12,5 % (*n* = 1)
Berufserfahrung	21,4 Jahre
Jahre EWS-Implementierung	3,6 Jahre

Die Ergebnisse der Runde 1 wiesen eine hohe inhaltliche Übereinstimmung auf. Aufgrund zahlreicher inhaltlicher Überschneidungen wurden die Projektphasen Vorbereitung und Planung zu einer gemeinsamen Phase zusammengeführt.

Im Ergebnis wurden 51 konsolidierte Items identifiziert (Zusatzmaterial, Tab. E2) verteilt auf 4 Projektphasen:

- Vorbereitung und Planung: 12 Items,
- Implementierung: 13 Items,
- Evaluation: 14 Items,
- Verankerung: 12 Items.

Diese Items bilden eine strukturierte Grundlage für die Ableitung praxisorientierter Implementierungsstrategien.

In der 2. Runde wurden alle 51 Items durch die 8 Delphi-Teilnehmenden auf einer Skala von 0 (= gar nicht relevant) bis 10 (= maximal relevant) bewertet. Alle Items erhielten einen medianen Bewertungswert von ≥ 7 Punkten und wurden damit als sehr wichtig für die erfolgreiche Implementierung von EWS eingestuft. Alle Bewertungen bis auf 5 wiesen einen IQR ≤ 2 auf, die höchste Streuung mit IQR = 4 wurde bei dem Item „Klare Verantwortlichkeiten und definierte Multiplikatoren“ ermittelt. Die höchsten Bewertungen entfielen auf die Items „Schulung und Onboarding für alle Berufsgruppen“ in der Phase Implementierung mit einem Medianwert von 10 Punkten (IQR = 1) gefolgt von „Interdisziplinäres Kernteam (Ärzte, Pflege, IT, QM, Controlling)“ in der Phase Vorbereitung und Planung mit 10 Punkten (IQR = 2).

In einem virtuellen Konsensustreffen wurden die zentralen Empfehlungen zur Implementierung anhand der Projektphasen ausgewählt. Zur Konsensusfindung zentraler Empfehlungen zur EWS-Implementierung wurde betont, dass die Einführung als Dienstanweisung der Geschäftsführung erfolgen und während der regulären Arbeitszeit geplant werden soll. Eskalationsstufen (Tab. 2) und ein entscheidungsbefugtes Rapid Response Team (RRT) müssen definiert, Prozesse abgestimmt und hochrisikoreiche Bereiche priorisiert werden. Die zentralen Empfehlungen sind:

1. Vorbereitung und Planung:
  Aufbau eines interdisziplinären Kernteams (z. B. Ärzt:innen, Pflegefachpersonen, IT, QM, Controlling) zur Steuerung des Projekts;
  Bildung eines zentralen Projektteams mit allen relevanten Berufsgruppen zur strukturierten Planung und Umsetzung;
  technische Integration von EWS2 und qSOFA in die klinischen Dokumentationssysteme, abgestimmt auf die bestehenden digitalen Strukturen;
  Festlegung von Alarmgrenzen, Triggern, Eskalationsstufen sowie Definition der beteiligten Endgeräte und Prozesse;
  klare Definition der Rekrutierung und Zuständigkeiten des Rapid Response Teams (RRT), z. B. durch bestehende Notfallteams;
2. Implementierung:
  Interprofessionelle Schulungs- und Onboardingformate für alle beteiligten Berufsgruppen zur sicheren und akzeptierten Anwendung des EWS;
  schrittweise Einführung – beginnend in Hochrisikobereichen mit vielen kritischen Ereignissen, anschließend sukzessive Ausweitung auf weitere Abteilungen;
3. Evaluation:
  offene Kommunikation über Ergebnisse, Erfolge und daraus abgeleitete Maßnahmen – mit transparenter Rückmeldung an alle Beteiligten;
  Einholung qualitativer und quantitativer Rückmeldungen zur Umsetzung und Nutzerfreundlichkeit des Systems;
  Dokumentation und Analyse von „lessons learned“, kontinuierliche Anpassung von SOP und strukturierte Evaluation zur stetigen Weiterentwicklung;
4. Verankerung:
  Sicherstellung langfristiger Verantwortung und Ressourcenplanung mit klar definierten Zuständigkeiten;
  Integration des EWS in bestehende Qualitätssicherungs- und Dokumentationssysteme (QM, PDMS) sowie in Fortbildungsangebote;
  Einbindung des EWS in Lehrcurricula und Einarbeitungskonzepte für neue Mitarbeitende – mit regelmäßiger Evaluation.

**Tab. 2:** Beispiel für Eskalationsstufen

Punktwert EWS2	Eskalation
0 Pkt.	Pflegefachperson überwacht Patienten weiter alle 12 h
1–4 Pkt.	Pflegefachperson überwacht Patienten alle 4–6 h
≥ 5 Pkt., bzw. 3 in einem Parameter	Pflegefachperson überwacht Patienten stündlich und informiert Arzt umgehend
	Arzt muss < 30 min Patienten sehen und evaluieren
≥ 7 Pkt.	Pflegefachperson überwacht Patienten kontinuierlich und informiert Arzt
	Arzt muss sofort Patienten sehen und evaluieren
	Pflegefachperson informiert ITS und RRT
	RRT kommt sofort und evaluiert Patienten

*EWS2* Early Warning Score 2, *ITS* Intensivstation, *RRT* Rapid Response Team

Die Empfehlungen betonen die interdisziplinäre/-professionelle Zusammenarbeit, digitale Integration, Schulung, Evaluation und nachhaltige Verankerung im Klinikalltag.

## Diskussion

Die Ergebnisse der modifizierten Delphi-Studie zeigen eine bemerkenswerte Einigkeit unter den Experten über die notwendigen strukturellen und prozessualen Bedingungen sowie die häufigsten Fallstricke bei der Einführung von EWS. Die Bewertungen unterstreichen die Bedeutung einer interprofessionellen und gut koordinierten Projektsteuerung und Implementierung mit allen Stakeholdern begleitet durch verbindliche Kommunikation, frühzeitige Evaluation und Verankerung in bestehende Klinikstrukturen. Diese Empfehlungen bieten eine fundierte Grundlage für Schulungskonzepte und Implementierungsstrategien im deutschsprachigen Raum.

In diesem modifizierten Delphi-Verfahren zur Implementierung von EWS haben nur 8 Personen teilgenommen. Vorausgesetzt dass die Rekrutierung und E‑Mail-Einladungen technisch einwandfrei verliefen und letztere nicht als Spam eingingen, könnte dieses Ergebnis zum einen auf eine geringe Anzahl von überhaupt verfügbaren Experten mit Erfahrung in der Implementierung von EWS hinweisen, aber auch durch andere Faktoren, wie Zeitmangel, unklare Zuständigkeiten usw., erklärt werden. Größere Gruppen sind anzustreben, wenn Dissens zu erwarten ist oder statistische Subanalysen geplant werden, beides ist hier nichtzutreffend [4]. Auch in der Literatur finden sich Delphi-Studien mit sehr kleinen Panels von 8–9 Experten zur personenzentrierten Übergabe in Notaufnahmen, zum Nutzen von Künstlicher Intelligenz im Krankenhaus oder zur Teamarbeit im Krankenhaus [9, 27, 32]. Sie zeigen, dass valide Ergebnisse möglich sind, wenn eine hohe inhaltliche Expertise und Konsensbildung vorliegen, wie auch in der vorliegenden Arbeit mit hohem Konsens mit einem IQR ≤ 2 in 90 % der Fälle in Runde 2, sodass eine 3. Runde nicht mehr notwendig war. Die Übertragbarkeit bleibt dadurch zwar eingeschränkt, die methodische Vorgehensweise ist jedoch in ähnlichen Kontexten anerkannt.

Die Ergebnisse decken sich mit der bisherigen Literatur [22]. Die Delphi-Studie hebt die Bedeutung eines interdisziplinären/-professionellen Kernteams (Medizin, Pflege, IT, QM, Controlling) als strukturelle Schlüsselbedingung hervor, dass die Implementierung von Anfang an begleitet und die Integration in das Klinikinformationssystem (KIS) sicherstellt. Dies deckt sich mit der Literatur, wonach die erfolgreiche EWS-Implementierung insbesondere in Hochrisikobereichen (z. B. Notaufnahme, Chirurgie, Post-ICU) an klare Zuständigkeiten und die digitale Infrastruktur gebunden ist [22]. Ein Vorteil strukturierter Steuerung ist die bessere Koordination komplexer Implementierungsaufgaben wie etwa Alarmgrenzdefinition oder Geräteausstattung [11]. Ein Nachteil kann jedoch in der Ressourcenbindung liegen, insbesondere wenn personelle oder finanzielle Mittel limitiert sind [18]. Vielversprechend sind Schulungsangebote mit u. a. interaktiven E‑learning-Kursen, interdisziplinären Trainings auf Stationen und Schulungen in Simulationszentren, bedürfen aber noch weiterer Forschung zur Effektivität [25, 28].

Im Mittelpunkt prozessualer Faktoren steht laut unseren Ergebnissen die Schulung und das Onboarding aller Berufsgruppen mit hoher Priorisierung (MW 9,3). Schulungen fördern sowohl das Wissen als auch das Vertrauen in die Anwendung von EWS, können jedoch bei unzureichender Qualität die Entwicklung kritischen Denkens behindern [6, 10, 17]. Vorteilhaft ist die Etablierung standardisierter Eskalationspfade und kontinuierlicher Feedbackzyklen („lessons learned“, SOP-Anpassungen), da diese Prozesse Transparenz und Reaktionsgeschwindigkeit erhöhen [7, 12]. Herausforderungen entstehen, wenn Schulungen selektiv oder zu spät erfolgen oder Evaluationsergebnisse nicht systematisch genutzt werden [25]. Zwar kann der Mehraufwand durch EWS pro Patient mehrere Minuten betragen, doch dieser Aufwand korreliert laut Studien mit einer verbesserten klinischen Entscheidungsqualität – insbesondere wenn digitale Tools (z. B. Dashboards, Red Flags) unterstützend eingesetzt werden [7, 8, 25, 30].

Die häufigsten Fallstricke betreffen mangelhafte Kommunikation, fehlende IT-Anbindung und unklare Verantwortlichkeiten. Die modifizierte Delphi-Studie identifiziert fehlende Zielbildkommunikation und ignoriertes Feedback als zentrale Fehlerquellen. Diese führen nicht nur zur Ablehnung durch Mitarbeitende, sondern erhöhen das Risiko der Alarmmüdigkeit, die auch in der Literatur als ein zentrales Hindernis gilt und für die sinnvolle Lösungen, wie ein zentrales Alarmmanagement, Anpassung der Grenzwerte und andere, zur Verfügung stehen [31]. Ein kritischer Punkt ist die voreilige Einführung ohne abgeschlossene Evaluation, was die Akzeptanz und Effektivität von EWS nachhaltig beeinträchtigen kann [28]. Demgegenüber ermöglichen gut evaluierte Roll-outs mit schrittweisem Feedbackmanagement eine kontinuierliche Optimierung [3]. Die Balance zwischen Standardisierung und Flexibilität ist dabei entscheidend, um auf lokale Gegebenheiten angepasst reagieren zu können, ohne die Systematik zu verlieren [28].

### Limitationen

Die modifizierte Delphi-Studie profitiert von der Einbindung erfahrener Praktiker aus dem IQM-Netzwerk, wodurch praxisnahe und realistische Empfehlungen generiert wurden. Die strukturierte Erhebung über mehrere Runden und das abschließende Konsensustreffen stärken die inhaltliche Validität. Die geringe Zahl der Teilnehmenden birgt ein Risiko für Verzerrungen und schränkt die Generalisierbarkeit ein; aufgrund der hohen Übereinstimmung zwischen den Runden ist der Effekt jedoch begrenzt, sodass die Schlussfolgerungen als vorsichtig, aber belastbar einzuschätzen sind.

Die Überrepräsentation von Pflegefachpersonen könnte die Perspektiven anderer Berufsgruppen verzerren; da sie jedoch zu den zentralen Stakeholdern im EWS-Prozess zählen, ist der Einfluss auf die Gesamtschlussfolgerungen gering, wenn auch nicht vollständig auszuschließen. Dennoch bietet die Studie wertvolle Einblicke in erfolgskritische Faktoren der EWS-Implementierung.

## Schlussfolgerungen

Die erfolgreiche Implementierung von EWS geht weit über die technische Einführung hinaus. Sie erfordert eine sorgfältige Vorbereitung, interprofessionelle Abstimmung, passgenaue Schulungskonzepte sowie eine kontinuierliche Evaluation und Rückmeldung. Besonders wirksam sind transparente Kommunikationsstrukturen und die feste Verankerung im klinischen Alltag. Krankenhäuser, die EWS einführen möchten, sollten die identifizierten Erfolgsfaktoren systematisch berücksichtigen, um eine nachhaltige, akzeptierte und wirksame Integration in bestehende Versorgungsstrukturen zu erreichen.

## Fazit für die Praxis

- Eine erfolgreiche EWS-Implementierung erfordert ein interdisziplinäres Projektteam, klare Verantwortlichkeiten und digitale Integration in bestehende Systeme.
- Interprofessionelle Schulungsformate und kontinuierliche Kommunikation über Ergebnisse stärken Akzeptanz und Anwendungssicherheit.
- Die langfristige Wirksamkeit hängt von der strukturellen Verankerung in Routinen, Qualitätsmanagement und Fortbildungsprogrammen ab.

## Supplementary Information

Tabelle E1: Anzahl der Vorschläge;
Tabelle E2: Bewertung zentraler Empfehlungen;
Tabelle E3: CREDES-Checkliste für Delphi-Studien

## Data Availability

Die erhobenen Datensätze sind im Artikel oder im Zusatzmaterial online zu finden.

## Footnotes

1 Es sind Personen aller Geschlechter gemeint. Zur besseren Lesbarkeit wurde die männliche Form gewählt.
