# Supplementary material for: Modified Delphi study about implementation of early warning scores
Source: Med Klin Intensivmed Notfmed. 2025 Oct 1;121(8):662–8. [Article in German] doi: 10.1007/s00063-025-01336-y (PMC13740071; doi:10.1007/s00063-025-01336-y)
Supplement: Supplementary file 1 [file 63_2025_1336_MOESM1_ESM.pdf]

Online Zusatzmaterial

Delphi-Studie zur Implementierung von Early Warning Scores

Nydahl P, Grossmann F, Franke V, Jeitziner MM, Krotsetis S, Kabir K, Lingenhag R, Melms T, Neuenfeldt FJ, Oroszy D, Regner F, Braun JP

Inhalt

Tabelle E1: Anzahl der Vorschläge
Tabelle E2: Bewertung zentraler Empfehlungen
Tabelle E3: CREDES-Checkliste für Delphi-Studien

Tabelle E1: Anzahl der Vorschläge

Projektphase	Strukturen	Prozesse	Pitfalls	Summe
Vorbereitung	43	52	36	131
Planung	40	35	29	104
Implementierung	31	32	39	102
Evaluation	22	30	28	80
Verankerung	32	27	29	88
Summe	168	176	161	505

Tabelle E2: Bewertung zentraler Empfehlungen

Projektphase	Median	IQR
**Phase 1 & 2: Vorbereitung & Planung**		
**Strukturen**		
Interdisziplinäres Kernteam (Ärzte, Pflege, IT, QM, Controlling)	10	2
Steuerungs- & Projektgruppe, inkl. Pilotbereich	9	3
Klare Verantwortlichkeiten & definierte Multiplikatoren	8	4
Projektauftrag & Rückendeckung (z. B. durch Letter of Intent)	7	2
**Prozesse**		
Auswahl und Anpassung eines EWS-Systems, inkl. Integration ins KIS	9	1
Erstellung von SOPs, Eskalationspfaden, Testläufen, Feedbackzyklen	8	1
Projektplan mit Meilensteinen und realistischer Zeit-/Ressourcenpla-nung	8	2
Klärung von Schulungsformaten, Kommunikationsstruktur und Zielbild	8	2
**Vermeidbare Fehler**		
Fehlende Kommunikation von Nutzen und Zielsetzung	9	2
Stakeholder zu spät eingebunden, Rollen unklar	8	2
Fehlende IT-Anbindung, Doppeldokumentation	8	2
Unrealistische Planung (keine Tests, keine Zeitpuffer)	7	2
**Phase 2: Implementierung**		
**Strukturen**		
Schulungsteam mit fachspezifischer Kenntnis & Erreichbarkeit	9	1
Projektteam mit festen Ansprechpartnern & Taskforce-Struktur	8	1
Kommunikations- & Feedbackstruktur vor Ort	8	1
Geeignete personelle Ausstattung	8	0
Geeignete Pilotstation mit technischer Ausstattung (z.B. SpotCheck Monitore)	8	0
**Prozesse**		
Schulung & Onboarding für alle Berufsgruppen	10	1
Begleiteter Go-Live mit Monitoring, Feedback, Huddles	8	1
Vorbereitung: Trigger definieren, Endgeräte konfigurieren	7	2
Laufende Qualitätssicherung, flexible Anpassung bei Bedarf	7	2
**Vermeidbare Fehler**		
Unklare Prozesse, keine Evaluation vor Go-Live	8	0
Feedback wird nicht strukturiert erfasst oder ignoriert	8	1
Technikprobleme, Infrastrukturmängel	8	2
Schulung nur selektiv oder verspätet	7	3
**Phase 3: Evaluation**		
**Strukturen**		
Erhebung klinischer Kennzahlen (z. B. Notrufe, ITS-Verlegungen, kritische Verläufe).	9	2
Feedbackkanäle & Anwendersprecher	8	3
Datenerhebung über IT, Logbuch, Dashboard	8	1
Entscheidungsgremium für Anpassung & Rollout	7	1
Integration in PDCA-Zyklus, Externe Vergleichsstrukturen	7	2
**Prozesse**		
Kommunikation der Ergebnisse, Erfolge & Handlungskonsequenzen	9	1
Lessons Learned, SOP-Anpassungen, strukturelle Evaluation	9	2
Qualitatives & quantitatives Feedback der Beteiligten	9	2

Kontinuierliche Analyse von Scores, Outcomes, Vollständigkeit	8	1
Go/No Go aufgrund der Pilotdaten entscheiden	7	1
**Vermeidbare Fehler**		
Ergebnisse nicht veröffentlicht oder inhaltlich ignoriert	8	1
Evaluation nur punktuell, ohne Einbindung aller Gruppen	8	1
Datenlücken, unpassende Auswertung, keine Nachvollziehbarkeit	7	1
Rollout vor abgeschlossener Evaluation	8	3
**Phase 4: Verankerung**		
**Strukturen**		
Verbindliche Verantwortung & Ressourcenplanung	9	0
Integration ins QM, PDMS, Fortbildungsprogramme	9	2
Zentrale Koordination & Rollout-Stufenplan	8	1
Monitoringstruktur & Train-the-Trainer-Netzwerk	8	1
**Prozesse**		
Verknüpfung mit Routinen (Visiten, Notfalltrainings, Audits)	9	2
Pflichtschulungen & Standardisierung von Checklisten/SOPs	8	2
Regelmäßige Evaluation, Fortschrittskommunikation	8	2
Strategische Weiterentwicklung & Einbettung in Klinikstrategie	7	1
**Vermeidbare Fehler**		
Kein Nachschulungsprogramm, keine Fortschreibung der SOPs	8	1
Ergebnisse nicht sichtbar gemacht, Erfolg nicht gefeiert	8	1
Verantwortung bleibt diffus, Rückmeldungen werden ignoriert	8	2
EWS bleibt Pilotprojekt ohne Verstetigung	8	3

Abkürzungen: EWS Early Warning Score; IQR Interquartilabstand; IT Informationstechnologie; ITS Intensivstation; PDCA Plan Do Check Act Zyklus; PDMS: Patient Data Management System; QM Qualitätsmanagement; SOP Standard Operation Procedure

Tabelle E3: CREDES-Checkliste für Delphi-Studien

CREDES-Item	Beschreibung	Umsetzung in dieser Studie	Seite
1. Ziel der Studie	Klar definierte Zielsetzung der Delphi-Erhebung.	Identifikation praxisrelevanter Bedingungen und Strategien zur erfolgreichen Implementierung von EWS in deutschen Krankenhäusern.	3-4
2. Design	Angabe des Studiendesigns, inklusive Anzahl der Runden.	Modifiziertes dreistufiges Delphi-Verfahren; Runde 3 entfiel aufgrund vorherigem Konsens.	4-5
3. Teilnehmende	Beschreibung der Teilnehmenden inkl. Einschlusskriterien.	Mitarbeitende aus IQM-Krankenhäusern mit erfolgreicher EWS-Implementierungserfahrung (n = 28).	4
4. Rekrutierung und Zustimmung	Verfahren zur Rekrutierung und Einholung informierter Zustimmung.	Einladung via IQM-Netzwerk; schriftliche Aufklärung und Einwilligung vor Teilnahme; Teilnahme freiwillig, aber nicht anonymisiert.	4
5. Fragebogenerstellung	Beschreibung der Fragenentwicklung und Kategorien.	In Runde 1: freie Nennung von Bedingungen in drei Kategorien (Strukturen, Prozesse, Pitfalls) entlang fünf Projektphasen der EWS-Implementierung.	4
6. Analyse und Zusammenfassung	Verfahren zur Aggregation, Clusterung und Darstellung der Antworten.	Inhaltsanalyse und KI-gestützte Zusammenfassung; Clustering durch Forschungsteam mit Validierung von Stichproben.	5
7. Konsensdefinition	Angabe, ab wann ein Konsens als erreicht gilt.	Konsens: Median-Bewertung ≥7 auf einer 11-Punkte-Skala (0–10), festgelegt vor Runde 2.	5

8. Feedback an Teilnehmende	Information, ob und wie Rückmeldung gegeben wurde.	Aggregierte Ergebnisse wurden den Teilnehmenden vor dem Konsensustreffen bereitgestellt.	6
9. Datenanalyse	Beschreibung der quantitativen und qualitativen Auswertung.	Quantitative Auswertung mit Excel (Mittelwerte, Mediane); qualitative Inhaltsanalyse offener Nennungen.	5-6
